# Lower limb perfusion during robotic-assisted laparoscopic radical prostatectomy evaluated by near-infrared spectroscopy: an observational prospective study

**DOI:** 10.1186/s12871-018-0567-8

**Published:** 2018-08-18

**Authors:** Kenichi Takechi, Sakiko Kitamura, Ichiro Shimizu, Toshihiro Yorozuya

**Affiliations:** 1 Matsuyama Red Cross Hospital, 1 Bunkyochou, Matsuyama City, Ehime Japan; 2 Department of Anesthesia and Perioperative Medicine, Ehime University Graduate School of Medicine, 454 Shitsukawa, Toon City, Ehime Japan

**Keywords:** Lower limb perfusion, Robotic prostatectomy, Lithotomy position

## Abstract

**Background:**

Decreased perfusion in the lower extremities is one of the several adverse effects of placing patients in a lithotomy or Trendelenburg position during surgery. This study aimed to evaluate the effects of patient positioning in lower limb perfusion patients undergoing robotic-assisted laparoscopic radical prostatectomy (RARP) using near-infrared spectroscopy (NIRS).

**Methods:**

This observation study comprised 30 consenting males with American Society of Anaesthesiologists physical status classes I and II (age range, ≥20 to < 80 years). Regional saturation of oxygen measurements was obtained using an INVOS™ oximeter (Somanetics, Troy, MI, USA). A NIRS sensor was positioned on the surface of the skin at the mid-diaphyseal region of the calf muscles (the gastrocnemius and soleus), over the posterior compartment, in the right lower leg. Regional saturation of oxygen (rSO_2_) was sampled during the following time points: before and 5 min after induction of anaesthesia (T0,T1); 5 min after establishment of pneumoperitoneum in a 0° lithotomy position (T2); 5 min after a 25° Trendelenburg position (T3); 30, 60, 90 and 120 min after pneumoperitoneum in a Trendelenburg position (T4, T5, T6 and T7, respectively); after desufflation in a supine position (T8); and after tracheal extubation (T9).

**Results:**

Lower limb perfusion evaluated by NIRS was increased after induction of anaesthesia and maintained during steep Trendelenburg positions in RARP patients with no risk for lower limb compartment syndrome (LLCS) (T0:65 ± 7.2%, T1:69 ± 6.1%, T2:70±:6.1%, T3:68 ± 6.7%, T4:66 ± 7.5%, T5:67 ± 6.9%, T6:68 ± 7.2%, T8:73 ± 7.2%, T9:71 ± 7.9%, respectively).

**Conclusions:**

Lower limb perfusion evaluated by NIRS was maintained during the RARP procedure. Correct patient positioning and careful assessment of risk factors such as vascular morbidity could be important for the prevention of LLCS during RARP.

## Background

Robotic-assisted laparoscopic radical prostatectomy (RARP) is one of the most commonly performed robotic-assisted surgical procedures worldwide. In RARP, the patient is placed in the lithotomy position and pneumoperitoneum is established, following which the patient is placed in the steep Trendelenburg position. The combination of lithotomy position, pneumoperitoneum, and steep Trendelenburg positioning may cause significant and potentially adverse cardiovascular, respiratory and neuro-physiological changes in the body [1]. The combination of these positions is also thought to decrease perfusion in the lower extremities by reducing blood pressure [2].

Lower limb compartment syndrome (LLCS) is a rare but serious complication of surgery in the lithotomy position, with an estimated incidence of one in 3500 cases [3]. However, in a recent study conducted in the United Kingdom, the incidence of the LLCS during RARP was reported to occur more frequently than previously recognised [4]. Tissue ischaemia with low perfusion is one of the most common causes of LLCS; nonetheless, the effect of the position of the patient on lower limb perfusion during RARP remains unclear. Near-infrared spectroscopy (NIRS) has been recommended as a method to detect perfusion deficits in peripheral tissues [5]. Therefore, the present study aimed to evaluate the effect of patient positioning during RARP by examining the changes in regional saturation of oxygen (rSO_2_) in the lower limbs using NIRS.

## Methods

Thirty adult males (American Society of Anaesthesiologists class I and II physical status) scheduled for RARP at the Ehime University Hospital, Toon, Ehime, Japan, from September 2013 to May 2014 were consecutively selected. Patients with previous episodes of peripheral vascular disease and those who were morbidly obese (body mass index > 35 kg/m^2^) were excluded from the study. Approval for the study was obtained from the institutional review board (registration number 1308010), and informed consent was obtained from the patients. The study was registered with the UMIN Clinical Trials Registry (000032897).

A standard anaesthetic technique was used, wherein routine monitoring of non-invasive arterial blood pressure, electrocardiogram (ECG) and oxygen saturation (SpO_2_) was conducted on arrival at the operating room. Anaesthesia was induced with propofol (1.5–2 mg/kg), remifentanil (0.15–0.3 μg/kg/min) and rocuronium bromide (0.8 mg/kg) and maintained using desflurane (4–6%), remifentanil (0.15–0.3 μg/kg/min) and rocuronium bromide (5–7 μg/kg/min). The ventilator settings were as follows: tidal volume, 8–10 mL/kg body weight; inspiratory:expiratory ratio, 1:2 or 1:1.5; inspired O_2_ fraction, 0.6 with air; and inspiratory fresh gas flow, 2 L/min. The respiratory rate was adjusted to 8–16 /min in order to maintain an end-tidal CO_2_ (ETCO_2_) pressure of 30–45 mmHg. After induction of anaesthesia, a 22-G radial artery catheter was inserted for blood sampling and to monitor the continuous arterial pressure and the minimally invasive cardiac output using FloTrac™ (Edwards LifeSciences, Irvine, CA, USA). The mean arterial pressure (MAP) was maintained above 60 mmHg.

A continuous dual-wavelength near-infrared spectrometer, INVOS™ oximeter (Somanetics, Troy, MI, USA), was used to determine the rSO_2_ measurements. A NIRS sensor was positioned on the surface of the skin, in the mid-diaphyseal region of the calf muscles (the gastrocnemius and soleus), over the posterior compartment of the right lower leg. For cerebral oximetry, NIRS sensors were placed unilaterally at least 2 cm above the eyebrow on the right side of the forehead according to manufacturer’s instructions. Both sensors were placed before the induction of anaesthesia during pre-oxygenation with 100% O_2_. A change of more than 8 percentage points in lower limb rSO_2_ was defined as clinically relevant [6].

For the lithotomy position, devices for vacuum-packed positioning and spreading the legs apart were used to absorb compressive forces, prevent uneven and potentially excessive pressure distribution and prevent excessive stretching or compression. After positioning, the compressive forces were measured using a portable pressure measuring device (PalmQ™, CAPE, Yokosuka, Japan). The positions were then adjusted and maintained in such a manner that the pressure values in the shoulder, hip and calf were below 30 mmHg.

The abdominal cavity was insufflated with CO_2_ gas at a pressure of 12 mmHg, following which the patients were placed in a 25° Trendelenburg position during surgery. The procedure was performed by a surgeon on a control table located away from the operating table, using the da Vinci robot surgical system (Intuitive Surgical, Sunnyvale, CA, USA).

MAP and rSO_2_ were recorded before the induction of anaesthesia. ETCO_2_, cardiac index and stroke volume variations were monitored after ventilation was controlled. The recorded values were sampled at the following time points: before induction of anaesthesia (T0); 5 min after induction of anaesthesia (T1); 5 min after establishing pneumoperitoneum with the patient in a 0° lithotomy position (T2); 5 min after the patient was placed in a 25° Trendelenburg position (T3); 30, 60, 90 and 120 min after establishment of pneumoperitoneum in the Trendelenburg position (T4, T5, T6 and T7, respectively); after desufflation in a supine position (T8); and after tracheal extubation (T9). The levels of haemoglobin, arterial partial pressure of oxygen (PaO_2_) and arterial partial pressure of carbon dioxide (PaCO_2_) were measured by arterial blood gas analysis at T1, T2, T3 and T7.

To calculate the sample size, the standard deviation of the baseline rSO_2_ of the first five patients (7.3) was used. Twenty-six patients with an error of 0.05 and a power of 80% were required to determine the changes in lower limb rSO_2_ (8 percentage points) in the current study. Assuming a dropout rate of 15%, 30 patients were recruited in this study.

All statistical analyses were performed using IBM SPSS Statistics 23 (SPSS Inc., Chicago, IL, USA). Continuous variables are presented as means and standard deviations. Repeated measured variables were analysed using a linear mixed model, with the patient indicator as random effect and time as fixed effects. Post-hoc analysis was performed with Bonferroni correction to adjust for multiple comparisons when the interactions between times were significant. All of the reported *p* values are two sided, and a *p* value < 0.05 was considered significant.

## Results

The demographics of the study participants are summarised in Table 1. No cases of LLCS were reported, none of the patients required blood transfusions, and all patients were satisfied with the surgical procedure.

**Table 1:** Patient demographics and operative variables

Variable	Mean ± SD
Age	67.7 ± 6.4
Weight (kg)	64.0 ± 5.6
Height (cm)	165.3 ± 5.7
Operation duration (min)	249.3 ± 47.4
Anaesthesia duration (min)	323.1 ± 48.1
Trendelenburg position duration (min)	220.6 ± 48.2
Pneumoperitoneum duration (min)	205 ± 47.9
Intake (ml)	1860 ± 371.1
Output (ml)	271.7 ± 216.7
Urine output (ml)	141 ± 118
Blood loss (ml)	192 ± 199

**Fig. 1:**
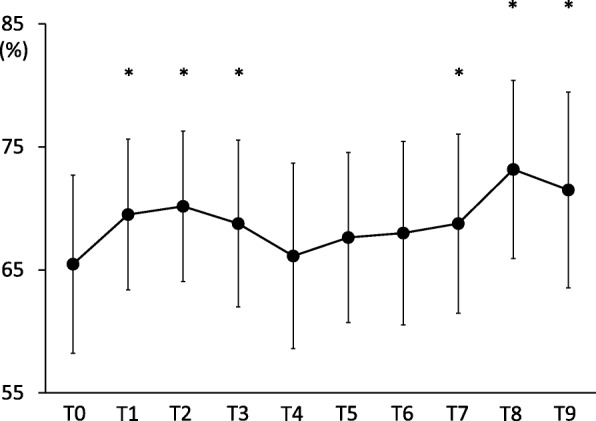
Mean lower limb rSO_2_ at each time point. Error bars denote the standard deviation. *P < 0.05 when compared with T0

Significant changes in rSO_2_ values (*p* < 0.01) were observed in the lower limb (Fig. 1) (T0:65 ± 7.2%, T1:69 ± 6.1%, T2:70±:6.1%, T3:68 ± 6.7%, T4:66 ± 7.5%, T5:67 ± 6.9%, T6:68 ± 7.2%, T8:73 ± 7.2%, T9:71 ± 7.9%, respectively). The values increased after induction of anaesthesia, establishment of pneumoperitoneum in the 0° lithotomy position, placing the patient in the Trendelenburg position (at 5 and 120 min), desufflation in the supine position and tracheal extubation when compared to the baseline values. No significant changes in rSO_2_ were noted between the time points during the Trendelenburg positions. Furthermore, the lower limb rSO_2_ values decreased by over 8 percentage points from the baseline after induction of anaesthesia in two patients and at 5 min after steep Trendelenburg positions in four patients. In addition, six patients demonstrated a decrease of more than 8 percentage points in lower limb rSO_2_ values during the steep Trendelenburg positions.

**Fig. 2:**
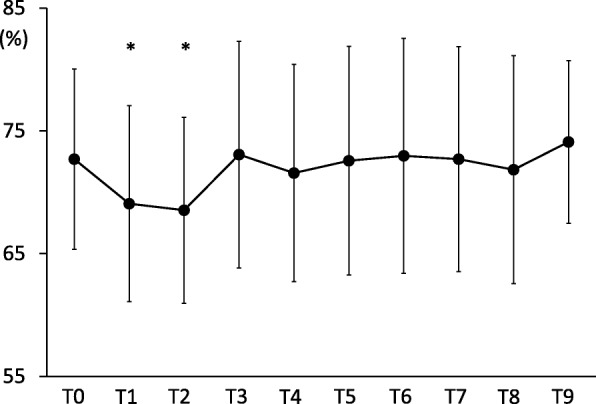
Mean cerebral rSO_2_ at each time point. Error bars denote the standard deviation. *P < 0.05 when compared with T0

Changes in cerebral rSO_2_ were also found to be statistically significant (*p* < 0.01) in the current study (Fig. 2). A decrease in cerebral rSO_2_ values was noted after induction of anaesthesia; however, the values returned to baseline during pneumoperitoneum at the Trendelenburg position. One patient presented with a decrease of < 75% from the baseline value 5 min after induction of anaesthesia (probably due to cerebral desaturation) and was treated with ephedrine (intravenous; 8 mg) to increase the cerebral perfusion pressure.

**Fig. 3:**
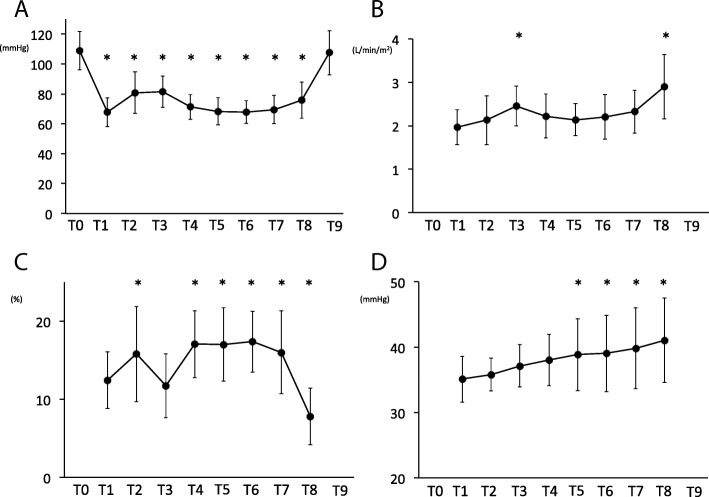
Mean blood pressure (**a**), cardiac index (**b**), stroke volume variations (**c**) and end-tidal CO_2_ values (**d**). Error bars denote the standard deviation. *P < 0.05 when compared with T0 or T1

MAP (A) decreased after induction of anaesthesia and continued to show a decreasing trend throughout the operation (Fig. 3). Cardiac index (B) increased at 5 min after a Trendelenburg position and after desufflation in a supine position when compared with the baseline values. Stroke volume variations (C) were temporarily recovered at 5 min after a Trendelenburg position but increased at other time points during the Trendelenburg position when compared with the values obtained at 5 min after induction of anaesthesia. ETCO_2_ (D) increased 60 min after establishment of pneumoperitoneum in a Trendelenburg position when compared to 5 min after induction of anaesthesia. Serum laboratory data related to the compartment syndrome (e.g. creatine kinase, lactate dehydrogenase) showed no changes when the preoperative values were compared with those on postoperative day one.

## Discussion

In the present study, lower limb perfusion evaluated by NIRS was found to be increased after induction of anaesthesia and maintained during the steep Trendelenburg position in RARP patients with no risk of LLCS.

LLCS is a rare but serious complication of RARP [7, 8]. The clinical diagnosis of acute compartment syndrome is difficult as the traditional use of the ‘6 Ps’ to diagnose LLCS has low specificity and is especially difficult to evaluate during the RARP procedure [9]. NIRS allows for the continuous monitoring of the tissue oxygenation of limbs at risk for ischaemia throughout the operative period. Lower limb NIRS was used to confirm the development of compartment syndrome after surgical cut-down for vascular access [10] and in a model of acute LLCS [6, 11]. To the best of our knowledge, this is the first clinical study to demonstrate the use of NIRS to assess lower limb perfusion during the RARP procedure.

There are several mechanisms for intraoperative LLCS. In RARP procedure, the patient’s legs are above the heart level in the lithotomy position and steep Trendelenburg position, and the blood pressure of the lower limb may decrease if these positions are held for prolonged periods. Decreases in blood flow may result in reduced oxygen supply within the compartment and lead to an inflammatory response, release of cytokines, capillary leakage and interstitial oedema. These responses increase the pressure within the compartment, escalating into a self-perpetuating cascade of worsening swelling. Additionally, direct external pressure from the calf support positioning device can also exacerbate the compartmental pressure. When the pressure rises above critical levels, intra-compartmental structures such as nerves, blood vessels and muscles are damaged. Obesity, peripheral vascular disease, prolonged console time and lack of experienced surgeons are increasing the risk of LLCS during RARP [4].

As reported previously, the cardiovascular factors that affect peripheral perfusion pressure, such as cardiac index, heart rate and stroke volume, were shown to decrease significantly after induction of anaesthesia in RARP patients [12]. Additionally, we observed that stroke volume variations, a surrogate marker of cardiac preload, were significantly increased during the Trendelenburg positions, except at 5 min after the Trendelenburg position. These findings suggested that the changes in cardiovascular physiology that occurred during RARP had negative effects on systemic perfusion and may affect cerebral rSO_2_. However, instead of this negative physiological change, lower limb rSO_2_ was increased after induction of anaesthesia and maintained during steep Trendelenburg positions in RARP patients. Volatile anaesthetics such as sevoflurane induced marked changes in muscle microcirculation, increase in tissue blood volume, decreased microvascular resistance and compliance, and decreased muscle oxygen consumption [13]. This favourable effect of volatile anaesthetics on microcirculation may be one of the mechanisms by which lower limb rSO_2_ is maintained during the RARP procedure. High inspiratory fractions of oxygen also help in increasing lower limb oxygenation. Moreover, hypercapnia is known to be beneficial for peripheral microcirculation [14]. Lower limb oxygenation may be compensated by increasing peripheral microcirculation. Therefore, limb positioning to avoid excessive compression force is crucial for the prevention of LLCS during RARP.

One of the limitations of this study is that it is not known whether maintenance of lower limb rSO_2_ during RARP is genuinely conducive for reducing the incidence of LLCS. Further studies are required to explore the perfusion pressure, microcirculation and clinical threshold of rSO_2_ values. Furthermore, different mechanisms of muscle microcirculation have been reported using different anaesthetics [15]. Therefore, the effects of various combinations of anaesthetics for lower limb perfusion during RARP must also be explored.

## Conclusions

In conclusion, lower limb perfusion evaluated by NIRS was maintained during the RARP procedure, probably by an increase in microcirculation. Hence, correct patient positioning and careful assessment of risk factors, such as vascular morbidity, might be important for the prevention of LLCS during RARP.

## Abbreviations

CI: Cardiac index
ECG: Electrocardiogram
ETCO_2_: End-tidal CO_2_
LLCS: Lower limb compartment syndrome
MAP: Mean arterial pressure
NIRS: Near-infrared spectroscopy
RARP: Robotic-assisted laparoscopic radical prostatectomy
rSO_2_: Regional saturation of oxygen
SpO_2_: Oxygen saturation
SVV: Stroke volume variations
